# Systematic Review of Group-Based Emotion Regulation Interventions for Autistic Children’s Socio-Emotional Competence

**DOI:** 10.1177/15394492251330507

**Published:** 2025-04-15

**Authors:** Sitii Hazwaanii Jasni, Fiona Graham, Elliot Bell, Valerie T. Y. Tan

**Affiliations:** 1 University of Otago, Wellington, New Zealand; 2 Massey University, Wellington, New Zealand; 3 University of Otago, Dunedin, New Zealand

**Keywords:** autism, socio-emotional competence, group-based emotion regulation, cognitive-behavioral therapy (CBT), systematic review

## Abstract

Autistic children often face challenges in socio-emotional competence (SEC). Group-Based Emotion Regulation Interventions (GBERs), involving parents and teachers, show potential for improving SEC while enhancing accessibility and cost-effectiveness. This systematic review evaluates the effectiveness of GBERs for autistic children. A systematic search of seven databases (2012–2022) identified studies on GBERs targeting SEC in autistic children aged 7–18 years. Studies were assessed for risk of bias. Seventeen studies were included. Cognitive-behavioral therapy (CBT)-based approaches were most prevalent, with some interventions incorporating play-based, mindfulness, or yoga-based approaches. Effective interventions featured components such as parent and teacher psychoeducation and skill reinforcement through homework. GBERs show promise in improving SEC among autistic children, although caution is warranted as some studies lack comparator groups. Occupational therapists may play a pivotal role in expanding GBERs’ access and supporting SEC development.

## Plain Language Summary

How Group Programs Help Autistic Children Improve Social and Emotional Skills With Support From Parents and Teachers

Autistic children often have difficulty understanding and managing emotions, which can make it harder for them to interact with others in daily life. This review looks at research on group-based programs that help improve social and emotional skills in autistic children. These programs often include parents and teachers in the process, which helps children practice these skills at home and in school. The research we reviewed focused on children aged 7–18 years, and we looked at studies from the past decade. We analyzed how effective these programs are and explored whether occupational therapists could adapt them to support autistic children. Cognitive-behavioral therapy (CBT)-based approaches were the most prevalent; play-based, yoga-based, and mindfulness approaches were also incorporated. Effective interventions included parent and teacher psychoeducation, along with skill reinforcement through structured homework activities. Overall, this review shows that group programs are helpful for improving social and emotional skills in autistic children. Occupational therapists could potentially play an important role in making these programs more widely available.

## Introduction

Socio-emotional competence (SEC) refers to an individual’s ability to understand, manage, and express social and emotional aspects of life effectively. This includes recognizing one’s own emotions, empathizing with others, and engaging in socially appropriate behaviors and relationships (Cai & Uljarević, 2021; Domitrovich et al., 2017; Goleman, 1996). For autistic children, SEC is crucial for interpreting and managing social and emotional cues. Difficulties in interpreting such cues may lead to social isolation, hindered learning, and low emotional self-awareness (Grace et al., 2022; Huggins et al., 2023). Higher SEC levels are associated with better academic performance and fewer social, emotional, and behavioral challenges (Li et al., 2023; McNair et al., 2025). Therefore, SEC is a pivotal skill in supporting the adaptive development of autistic children. Challenges with SEC significantly impact their participation in life domains, such as home life, friendship, play, and leisure (Hilton et al., 2023). Addressing SEC enhances autistic children’s social participation (McNair et al., 2025) and occupational engagement (Egan & Restall, 2022). Parents and teachers play a critical role in emotional development by acting as “emotional coaches” and reinforcing SEC skills during teachable moments (Cao et al., 2022; Havighurst et al., 2019; Lauw et al., 2014). Successful parent-teacher partnerships further support generalization of skills across settings (McIntyre et al., 2022, 2023).

Traditionally, interventions addressing SEC difficulties were delivered individually. However, group-based interventions are increasingly recognized for their effectiveness in enhancing social skills (Ranjan et al., 2014), adaptive behavior, quality of life and fine motor skills (Hirschmann et al., 2023). These interventions provide collaborative environments where peer interactions improve on children’s emotional competencies, reduce isolation for both children and parents, and promote social learning (Hirschmann et al., 2023). They also facilitate better family communication, reduce parental stress, and offer practical benefits, such as reduced wait times and costs, and improved access to therapeutic services (McKenzie et al., 2019). In addition, families may benefit from mutual support (McKenzie et al., 2019; Sterman et al., 2023; Tisna Yanti et al., 2022). Preliminary research suggests that group-based interventions are as effective as individual therapies in improving socio-emotional skills in autistic children, thus highlighting the need for further research (Hirschmann et al., 2023). A systematic review concluded that parent-mediated interventions enhance emotion regulation in younger autistic children (Hendrix et al., 2022). However, their effectiveness for older autistic children, particularly those aged 7–18 years, with parental and teacher involvement, remains unclear. Furthermore, research on group-based delivery methods for supporting emotion regulation in this age group is also limited. Addressing the developmental and SEC needs of school-aged children and adolescents, a rather underrepresented group in SEC research, warrants further investigation (Ohl et al., 2013; Portela-Pino et al., 2021).

Occupational therapists (OTs) are key members of multidisciplinary teams, supporting the SEC of autistic children and providing well-being strategies for parents and teachers (Moosa et al., 2023). SEC is critical for autistic children’s occupational participation in play, self-care, and social relationships (Fischer et al., 2023; Hilton et al., 2023; Rodger & Ziviani, 2006), which are central outcomes in OT practice. OTs’ expertise in fostering children’s self-regulation and family engagement is highly relevant to developing SEC skills (Ayres, 1977; Ayres & Robbins, 2005; Haradon et al., 1994; Mahler et al., 2022; Miller et al., 2007; Mills et al., 2020; Tomchek & Dunn, 2007). However, the feasibility of delivering group-based emotion regulation (GBER) interventions through OT services remains unclear.

This systematic review examines the effectiveness, characteristics, target populations, and outcomes of GBER interventions in improving SEC for autistic children aged 7–18 years.

## Methods

This systematic review was pre-registered with the International Prospective Register of Systematic Reviews (PROSPERO: CRD42022339701). The methodology followed the Preferred Reporting Items for Systematic Reviews and Meta-Analyses (PRISMA) guidelines (Page et al., 2021).

### Search Strategy and Selection Criteria

The first author conducted a systematic search for articles across the following databases: Scopus, Web of Science, MEDLINE (Ovid), CINAHL (EBSCO), PsycINFO (Ovid), Complete Psychology & Behavioral Sciences Collection (EBSCO), and PubMed. These databases were selected for their multidisciplinary coverage (Scopus, Web of Science, MEDLINE, PubMed), focus on mental health literature (PsycINFO, Complete Psychology & Behavioral Science Collection) and emphasis on allied health (CINAHL). The searches were conducted on April 2, 2022, and repeated in November 2022. The alerts for saved searches were monitored regularly until January 31, 2024, to identify additional publications. The publication range was limited to 2011–2022 with no language exclusion. The search strategy used keywords and subject headings relevant to autism spectrum disorder (e.g., autis*, Asperger*, “pervasive developmental disorder”), emotion regulation intervention (e.g., “emotion* regulation, “mood regulation,” “affect* regulation”), program (e.g., intervention*, treatment*, module*), parent (e.g., parent*, caregiver*, famil*), and teacher (e.g., teach*, educat*, tutor*, “school counselor*,” “teach* aide*”). Synonyms for autism spectrum disorder were based on the Diagnostic and Statistical Manual for Mental Disorders (5th ed.; *DSM-5;* American Psychiatric Association, 2013). Search terms were also informed by systematic reviews from Cibralic et al. (2019) and Hendrix et al. (2022). Details are provided in Supplemental Table 1. A time filter was applied for 2012–2022. Articles were included if they met criteria outlined in Table 1. Eligible studies included peer-reviewed journals, and quantitative studies such as randomized controlled trials (RCTs), feasibility studies, pilot studies, pre- and post-test studies, quasi-experimental designs (QEDs), systematic reviews and meta-analyses. The search strategy initially considered articles published in multiple languages, including English, Malay, Mandarin, Tamil, Arabic, and Māori to ensure a comprehensive review. Abstracts in non-English languages were translated using Google Translate.

**Table 1:** Inclusion and Exclusion Criteria.

Criteria	Inclusion	Exclusion
Population	Having a diagnosis of ASD including autistic disorder, Asperger’s Syndrome, and pervasive developmental disorder-not otherwise specified, as defined by *DSM*-5, ICD-10, confirmed using standardized tools such as the ADOS and the ADI-R. Without ID or with mild ID, as assessed using standardized measures of intellectual and adaptive functioning. Aged between 7 and 18 years (inclusive)^a^.	Having other developmental disabilities such as attention deficit hyperactivity disorder, Down syndrome, fragile × syndrome, global developmental delay, depressive and anxiety disorder, conduct disorder, fetal alcohol spectrum disorders, reactive attachment disorder, sleep disorders. Having other physical disabilities such as cerebral palsy, hearing loss, Tourette’s syndrome/disorder, muscular dystrophy, developmental coordination disorder, locomotor impairment, or hearing loss. Moderate to profound ID.
Intervention	Studies were included if the intervention included: The teaching of strategies to recognize one’s own and others’ emotions and managing emotions. Delivery of interventions to groups (multiple children and families present) but may have had some individual sessions. Active parent and/or teacher involvement to support skill generalizations to daily life across settings (i.e., at home or school); and Delivery over more than one session.	Studies were excluded if the intervention: Was only delivered to individuals without active parent and/or teacher involvement. Was delivered as a one-off event. Was a lab-based physiological approach (e.g., electroencephalography-based neurofeedback training).
Comparators	No criteria	No criteria
Primary study outcome	Indices of emotion regulation, and/or socio-emotional competence, including social engagement (e.g., parent-child social engagement)	Any other outcome such as driving skills, communication skills, prosocial behaviors (e.g., helping, sharing, consoling, comforting, cooperating, and protecting someone from harm)
Secondary study outcome	Functional skills (e.g., adaptive skills, activity of daily living, self-care, feeding, functional mobility, household tasks, education-related tasks, i.e., school participation, community participation, personal safety, play activities) Communication skills Executive functioning	None

*Note.* ASD = autism spectrum disorder; *DSM*-5 = Diagnostic and Statistical Manual of Mental Illness Fifth Edition; ICD-10 = International Statistical Classification of Diseases and Related Health Problems 10th Revision; ADOS = Autism Diagnostic Observation Schedule; ADI-*R* = Autism Diagnostic Interview-Revised; ID = intellectual disability.

a Studies with reported age ranges extending beyond 7–18 years (e.g., 6–12 years) were included if the mean age of participants fell within the target range of 7–18 years. This approach ensures a comprehensive evaluation and account for variability in reported age range across studies.

**Figure 1.**
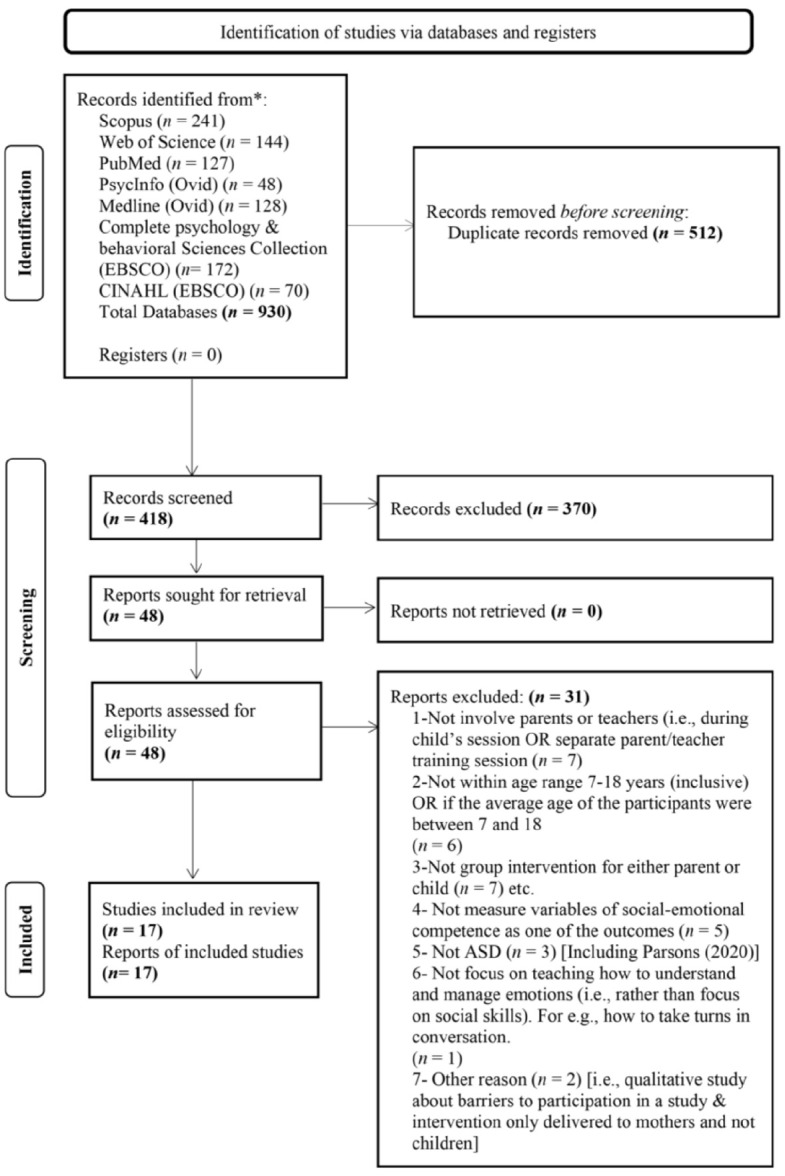
PRISMA Flowchart.

All four authors independently applied the eligibility criteria for reviews at the title, abstract and full-text screening stages. The first author downloaded all retrieved records into EndNote 20 and removed duplicates. The inclusion and exclusion criteria were then piloted on 30 randomly selected publications. In case of conflict, the disagreements were resolved through discussion, achieving 100% consensus. The first author independently screened all titles and abstracts, while the remaining three reviewers each screened one-third of the records, ensuring all publications dual independent review of all publications. Full texts were retrieved by one reviewer (S.H.J) and distributed among all reviewers for further screening. Reasons for excluding ineligible studies were recorded by the first author. This rigorous process ensured transparency and reproducibility. The PRISMA Flowchart summarizes this process (see Figure 1).

### Evaluation of Methodological Quality

The review initially followed the Cochrane Handbook methodology (Higgins et al., 2024) for assessing methodological quality. However, due to the significant heterogeneity in the dataset—comprising a mix of RCTs and non-RCTs with diverse interventions and variable reporting standards—the Joanna Briggs Institute (JBI) criteria were adopted for greater flexibility.

The JBI critical appraisal tools for randomized control trials (RCTs) and QED Studies (Aromataris et al., 2024; Porritt et al., 2014; Tufanaru et al., 2020) were employed to evaluate methodological quality and risk of bias. This dual approach aligns with recommendations for using robust tools across study designs (Kennedy et al., 2019). Seventeen studies met the inclusion criteria and were subjected to risk-of-bias assessment. Two independent reviewers conducted the assessment (S.H.J. and F.G.), with criteria scored “yes” (1 point), or “no” (0 point), or “unclear” (0 point) (Aromataris et al., 2024; Tufanaru et al., 2020). Discrepancies were resolved through discussion, achieving 100% consensus. The median score served as the threshold for categorizing studies as “low risk of bias,” a robust approach for heterogenous datasets (McGrath et al., 2020; Wan et al., 2014). This ensures transparent categorization, avoiding arbitrary exclusions while emphasizing rigor (Viswanathan et al., 2017). No studies were excluded based on critical appraisal scores, as the aim was to provide a comprehensive overview of the literature on the GBER to guide future research. See Supplemental Tables 2 and 3. The table was adapted from Watroba et al. (2023).

### Data Extraction and Synthesis

The first author extracted descriptive data, including general information (author, year, title, journal), methods (i.e., diagnosis, design, sample size, setting, country, follow-up), participant characteristics (mean age, gender, ethnicity), intervention details (intervention/program name, frequency, duration, content, adherence to intervention protocol, fidelity measure, mode of delivery such as face-to-face, online, type of parental/teacher involvement), and analytical data (outcome measures, and overall risk of bias).

Summaries of the GBER interventions are presented in Tables 3 and 4. Since the assumption of homogeneity (Aromataris et al., 2024; Brown & Richardson, 2017; Higgins et al., 2024) was not met, due to significant differences across the RCTs, including protocols diversity and inconsistent outcoming reporting, a meta-analysis was not conducted. Only two RCTs were categorized as low risk of bias (Parsons et al., 2019b; Soorya et al., 2015), Consequently, a narrative synthesis was performed. This systematic review did not require institutional review book/ethics approval, as it involved secondary analysis of published literature without direct interaction with human subjects.

## Results

### Study Selection

Initial searches across all databases identified 930 articles. After removing 512 duplicates, 418 articles remained eligible for title and abstract screening. Full-text article screening was conducted on 48 studies, with 31 articles excluded for not meeting the review criteria. No additional studies were identified through reference list searches of the included studies. Seventeen articles were included in the final review. See Figure 1 for details.

### Study Characteristics

Seventeen studies met the inclusion criteria, as summarized in Table 2. These studies varied in population, design, and intervention characteristics, focusing on SEC in autistic children.

**Table 2:** Study Characteristics of Included Studies.

Article, design, country & setting	Diagnosis/comorbidity	Sample size, µ *=* age (*SD*), age range	Gender/ethnicity	IQ level (*SD*) & measure name
(Beaumont et al., 2015) NRCT; two-group pre-post-test; Australia; mainstream school	HFASD: AS, HFA, PDDNOS Comorbidity: ADHD (21.7%); Anxiety (18.8%); Depression (5.6%); ODD (4.2%); SPD (4.3%); Speech and Language Impairments (4.3%)	*N* = 69; Condition 1 (*n* = 35), µ = 9.82 (1.63); Condition 2 (*n* = 34), µ = 9.25 (1.48) 7–12 y.o.	Condition 1 (94.3% male); Condition 2 (91.2% male); NR	Condition 1: FSIQ 99.41 (13.36); VIQ 97.05 (11.48); PIQ 104.00 (18.60) Condition 2: FSIQ 109.21 (15.24); VIQ 107.21 (14.13); PIQ 108.52 (17.85) WASI-II
(Einfeld et al., 2018) NRCT; two-group pre-post test Australia; School	ASD without ID	*N* = 84; Tx (*n* = 70), µ = 10.9 (1.4); TAU (*n* = 14), µ = 10.3 (1.6) 8.2–14.6 y.o.	89% male; NR	Tx: PIQ = 94.1 (19.2) TAU: PIQ 99.8 (19.4) NS
(Lopata et al., 2017) NRCT; single-group pre-post-test (feasibility); USA; College campus (group rooms)	HFASD: autism, AS, PDDNOS	*N* = 44; µ = 9.09 (1.70) 7–12 y.o.	93.2% male; Caucasian (88.6%); African American (2.3%); Latino (2.3%); Asian (2.3%); Other (4.5%)	FSIQ 108.61 (14.54); VCI IQ 108.39 (15.33); PRI IQ 106.94 (17.62) WISC-V
(Lopata et al., 2021) RCT; USA; College campus (outpatient setting)	ASD without ID	*N* = 88; Tx (*n* = 44), µ = 9.77 (1.76); WLC (*n* = 44), µ = 9.57 (1.73) 7–12 y.o.	Tx: 86% male; WLC: 84% male Caucasian (80.7%)	Tx: FSIQ 104.06 (13.13); VCI 104.63 (12.13); PRI 102/58 (15.48) WLC: FSIQ 105.22 (12.29); VCI 102.31 (12.63); PRI 107.16 (16.04) WISC-V
(Lopata et al., 2012) NRCT; single-group pre-post-test (feasibility); USA; School	HFASD	*N* = 12; µ = 7.72 (1.18) 6–9 y.o.	83% male; Caucasian (91.7%); Biracial (8.3%)	FSIQ 102.31 (16.01) WISC-V
(Lopata et al., 2019) Cluster-RCT; USA; Public elementary schools	ASD without ID	*N* = 103; Tx (*n* = 52), µ = 8.65 (1.29); SAU (*n* = 51), µ = 9.01 (1.45) 6–12 y.o.	Tx: 90.4% male; SAU: 92.2% male Children: Caucasian (96.1%); Staff: Caucasian (93%–100%)	Tx: FSIQ 103.82 (12.94); VCI IQ 103.04 (14.39); PRI IQ 103.82 (15.82) SAU: FSIQ 100.94 (14.84); VCI IQ 100.21 (14.07); PRI IQ 101.50 (16.59) WISC-V
(Lopata et al., 2015) NRCT; single-group pre-post design (feasibility); USA; Community agency	HFASD: AS, HFA, PDDNOS	*N* = 28; µ = 8.39 (1.03) 7–10 y.o.	89% male; Caucasian (92.9%); Asian American (3.6%); Other (3.6%)	Full IQ 105.79 (16.27) VCI IQ 106.60 (15.75) PRI IQ 103.87 (17.06) WISC-V
(Parsons et al., 2019a) Mixed-method; single-group pre-post (feasibility); Australia; Clinic	ASD without ID	*N* = 20; ASD (*n* = 10), µ = 8.7 (1.72); Playmates (*n* = 10), µ = 9.3 (1.98) 6–12 y.o.	ASD: 90% male; Playmates: 50% male NR	NR
(Parsons et al., 2019b) RCT; Australia; Clinic	ASD without ID	*N* = 136 ASD (*n* = 68): Tx: µ = 8.6 (1.38); WLC: µ = 8.4 (1.36) Playmates (*n* = 68): Tx: µ = 8.6 (1.83); WLC: µ = 8.4 (1.48) 7–10 y.o.	ASD: Tx: 93% male; WLC: 82% male Playmates: Tx: 44% male; WLC: 57% male NR	NR
(Ratcliffe et al., 2014) NRCT; two-group pre-post-test; Australia; mainstream schools	ASD without ID	*N* = 217; Tx (*n* = 106), µ = 9.40 (1.47); CTR (*n* = 111), µ = 9.57 (1.23) 7–13 y.o.	Tx: 90% male; CTR: 89.6% male NR	NR
(Ratcliffe et al., 2019) NRCT; two-group pre-post-test; Australia; various schools	ASD and Mild ID	*N* = 85; Tx (*n* = 52), µ = 9.41 (1.48); WLC (*n* = 33), µ = 9.12 (1.36) 7–13 y.o.	Tx: 83% male; WLC: 97% male NR	IQ range 50–55 to 70 NS
(Salem-Guirgis et al., 2019) NRCT; within-subject repeated measures design; Canada; Community setting	ASD Youth without ID	*N* = 23 parent-youth dyads Youth: µ = 15.65 (2.57); 12–23 y.o.; Parent: µ = 50.05 (5.25); 40–59 y.o.	Youth: 82.6% male; Parents: 13.0% male Youth: White/Caucasian (71.4%); Parents: White/Caucasian (71.4%)	FSIQ 103.96 (12.98) WISC-IV
(Soorya et al., 2015) RCT; USA; Outpatient autism treatment program	ASD without ID Comorbidity: ADHD, Anxiety, Depression	*N* = 69; Tx (*n* = 35), µ = 10.05 (1.27); CTR (*n* = 34), µ = 9.87 (1.32) 8–11 y.o.	Tx: 85.7% male; CTR: 84.38% male White (43%); Black (21%); Hispanic (26%); Asian (1%); Other (9%)	Tx: FSIQ 94.86 (17.34); VIQ 97.91 (16.70); NVIQ 100.5 (18.22) CTR: FSIQ 93.72 (16.79); VIQ 96.44 (15.20); NVIQ 98.97 (16.11) WISC-IV
(Stichter et al., 2012) NRCT; single-group pre-post; USA; interdisciplinary diagnostic & outpatient treatment center (classroom setting)	HFASD: autism, AS, PDDNOS, ASD	*N* = 20; µ = 8.77 (1.33) 6–10 y.o.	95% male NR	FSIQ 99.30 (15.18) NS
(Tanksale et al., 2021) RCT; Australia; university-affiliated program (community setting)	ASD without ID Comorbidity: ADHD, anxiety disorders, parent-reported anxiety, parent-reported sleep problems, medical (e.g. respiratory, cardiac, and allergies)	*N* = 67; Tx (*n* = 34), µ = 9.42 (1.34); WLC (*n* = 33), µ = 9.46 (1.38) 8–12 y.o.	Tx: 67.7% male; WLC: 60% male NR	Tx: FSIQ 94.21 (16.35); VCI 101 (4.24) WLC: FSIQ 95.83 (17.10) VCI 95.00 (NR) WASI-II
(Thomeer et al., 2012) RCT; USA; College campus (group rooms)	HFASD: AS, PDDNOS, HFA	*N* = 35; Tx (*n* = 17), µ = 9.24 (1.64); WLC (*n* = 18), µ = 9.39 (1.91) 7–12 y.o.	Tx: 82.4% male; WLC: 88.9% male Caucasian (80%); African American (5.7%); Hispanic (2.9%); Asian American (2.9%); Other (8.6%)	Tx: FSIQ 104.26 (14.13) WLC: FSIQ 103.42 (13.26) WISC-IV
(Thomson et al., 2015) NRCT; single-group pre-post-test (feasibility); Canada; community setting	ASD without ID	*N* = 14; µ = 10.40 (1.30) 8–12 y.o.	92.9% male NR	FSIQ 107.00 (11.54) WASI-II

*Note.* HFASD = high-functioning autism spectrum disorder; AS = Asperger’s syndrome; HFA = high-functioning Autism; PDDNOS = Pervasive Developmental Disorder-Not Otherwise Specified; ASD = Autism spectrum disorder; ID = Intellectual disability; CTR = Control group; Tx = Treatment group; SAU = Services-as-Usual; WLC = Waitlist-control; NR = Not reported; IQ = Intelligence quotient; FSIQ = Full Scale IQ; VIQ = Verbal IQ; PIQ = Performance IQ; VCI = Verbal Comprehension Index; PRI = Perceptual Reasoning Index; NVIQ = Nonverbal IQ; SPD = Sensory processing disorder; ODD = Oppositional defiant disorder; ADHD = Attention-deficit/hyperactivity disorder; NS = not specified; NRCT = non-randomized controlled trial; RCT = randomized controlled trial; WASI-II = Wechsler Abbreviated Scale of Intelligence—Second Edition; WISC-IV = Wechsler Intelligence Scale for Children—Fourth Edition; WISC-V = Wechsler Intelligence Scale for Children—Fifth Edition. Studies with reported age ranges extending beyond 7–18 years (e.g., 6–12 years, 6–10 years) were included if the mean age of participants fell within the target range of 7–18 years, as outlined in the inclusion criteria. This approach ensures a comprehensive review of interventions, particularly for studies authored by the same team with varying reported age ranges (e.g., Lopata et al., 2012, 2019; Parsons et al., 2019a, 2019b).

### Study Design

Among the 17 included articles, six were RCTs, 10 were quasi-experimental studies, and one employed a mixed-methods approach. Details of the study designs are provided in Table 2.

### Participants

The 17 studies included a total of 1,218 participants. Six studies focused on children with high-functioning autism spectrum disorder (HFASD) (Beaumont et al., 2015; Lopata et al., 2012, 2015, 2017; Stichter et al., 2012; Thomeer et al., 2012), one on children with ASD and mild intellectual disability (ID) (Ratcliffe et al., 2019) and the remaining ten on ASD without ID (Einfeld et al., 2018; Lopata et al., 2019, 2021; Parsons et al., 2019a, 2019b; Ratcliffe et al., 2014; Salem-Guirgis et al., 2019; Soorya et al., 2015; Tanksale et al., 2021; Thomson et al., 2015). Participants ranged from 6 to 23 years old, with six studies focusing on children older than 12 (Beaumont et al., 2015; Einfeld et al., 2018; Lopata et al., 2015; Ratcliffe et al.,2014, 2019; Salem-Guirgis et al., 2019). The mean age was 9.36 years. Male participants (*n* = 1,021) significantly outnumbered females (*n* = 197). Ethnicity data were reported in eight studies, with most participants identified as White/Caucasian (*n* = 320), followed by Hispanic/Latino (*n* = 20), African American/Black (*n* = 18), Other (*n* = 12), Asian (*n* = 4), and biracial/mixed race (*n* = 1). See Table 2.

### Intervention Characteristics

Interventions varied in approach, components, dose, group size, delivery mode, program facilitators, protocol adherence and fidelity monitoring, parent and/or teacher involvement, and accessibility. See Table 3.

**Table 3.** Structure and Content of GBER Interventions.

Article & accessibility	Intervention	Facilitators & protocol adherence/fidelity	Parent/teacher involvement & homework
(Beaumont et al., 2015) Require purchase	SAS (Beaumont, 2010): 10-wk F2F school-based prog. for children with HFASD (3 children), 90 minutes/sess. Structured (manualized) vs. unstructured. No comparator; pre-post design. CBT approach: focus on ER, social interaction, and behavior at school/home. SAS computer game, classroom activities. Behavioral strategies: positive reinforcement.	SPED, counselors, support staff, classroom teachers; Training DVD, Wk. phone/email support; Self-report checklist (75% to 95% adherence).	Parent: Separate training sess. (workbook, info sess.); Teacher: In-sess. facilitation (wk. tips sheets, sess. facilitation). HSD.
(Einfeld et al., 2018) Require purchase	SAS (Beaumont, 2009): 10–13 wk F2F school-based prog. for ASD (3–6 children), 90 minutes/sess. Comparator (TAU). CBT approach: social skills, ER, and PS. Interactive tools (SAS computer game, “code cards”).	Teachers trained in the SAS prog., supported by teacher aides; Checklists; VR; Independent coding (94% completion for child sess., 97% for parent sess.).	Parent: Separate training sess. (4 sess. × 2 hours, phone calls at 3 & 6mo); Teacher: In-sess. facilitation (wk. tip sheets). HSD.
(Lopata et al., 2017) NS	CSBI-MAXout (Lopata et al., 2010): 18-wk F2F prog. for HFASD (4 children), 2x/wk, 90 minutes/sess. No comparator; pre-post design. CBT approach: comprehensive OP psychosocial treatment. Focus on social communication, facial emotion recognition, non-literal language. Behavioral system to reduce ASD symptoms and increase skills.	UG & grad. Students (supervised); 100% on manual mastery exam; 5-day training; High fidelity (95% skills group, 94% therapeutic activities, 49% sess. observed).	Parent: Separate training sess. (six 90-minute over 18 wk); Teacher: NR. HW assigned after each sess. to reinforce skills.
(Lopata et al., 2021) NS	CSBI-MAXout (Lopata et al., 2010): 18-wk F2F prog. for ASD children without ID (4 children), 2x/wk, 90 minutes/sess. RCT; WLC. CBT approach: OP psychosocial treatment. Focus on social/social-communication, facial emotion recognition, non-literal language, interest expansion, and reducing ASD symptoms. Comprehensive behavioral system: points, feedback, response-cost.	Staff clinicians (UG & grad. students); 100% on manual mastery exam; 5-day training; High fidelity (96% adherence skills groups, 60% sess. observed)	Parent: Separate training sess. (six 90-minute sess. for 18 wk); Teacher: NR. HW assigned to practice skills.
(Lopata et al., 2012) SchoolMAX	CSBI-SchoolMAX (Lopata et al., 2010): 10-mo F2F school-based prog. for HFASD (3–6 children), 60–210 minutes/wk. No comparator; pre-post design. CBT & ABA approach. Multicomponent intervention: task analysis, emotion recognition, behavioral regulation, independence, and peer training. Focus on social competence and reducing ASD symptoms.	School staff and research team members (SLP, SW, CP, teachers); Fidelity ≥87 for all components.	Parent: Separate training sess. (mo PT sess.; 60–90 minutes each); Teacher: In-sess. facilitation. HW assigned through IDN and PT.
(Lopata et al., 2019) SchoolMAX	CSBI-SchoolMAX (Lopata et al., 2010): 9-mo F2F school-based prog. for ASD children without ID (1–5 children), 160–210 minutes/wk. Control: SAU. CBT & ABA approach. Multicomponent, manualized intervention. Focus on social competence, emotion recognition, and reducing ASD symptoms.	School staff (CPs, SLPs, SWs, classroom aides, and teaching assistants); 30-hour manualized training; High fidelity (≥92% adherence for all components).	Parent: Separate training sess. (monthly; 60–90 minutes each); Teacher: In-sess. Facilitation (subcomponent intervention delivery & IDN). HW assigned through PT.
(Lopata et al., 2015) Institute for Autism Research	CSBI-Community-SummerMAX (Lopata et al., 2010): 5-wk F2F community-based prog. for HFASD (7 children), 5 days/wk, 5x70 minutes/sess. No comparator; pre-post design. CBT approach. Manualized intervention: social skills, face-emotion recognition, non-literal language, interest expansion. Behavioral system: response-cost, IDN.	UG & grad. students (supervised by clinical directors); Standardized checklists; Prior training of clinical directors; High fidelity (≥ 92% for all components).	Parent: Separate training sess. (90 minutes/wk); Teacher: NR. Wk. home assignments.
(Parsons et al., 2019a) May need to contact author	Peer-to-peer play-based intervention (Wilkes-Gillan et al., 2016): 10-week F2F clinic-based prog. for ASD without ID (2 children per group; ASD + TD), 1 sess./wk, 60 minutes/sess. No comparator; pre-post design. Play-based approach. Focus on pragmatic language and social-emotional regulation. Peer modeling and VM.	SLP or OT; Intervention manual; Therapist training; Video feedback; Fidelity (% NS).	Parent: Separate training sess. (Observed sess. & facilitated home practice); Teacher: NR. Parents read manual, children watch DVD and host playdates.
(Parsons et al., 2019b) May need to contact author	Peer-to-peer play-based intervention (Wilkes-Gillan et al., 2016): 10-wk F2F clinic-based prog. for ASD without ID (2 children/group; ASD + TD), 1 sess./wk, 60 minutes/sess. RCT; WLC.	SLP or OT; Intervention manual Therapist training; Fidelity: therapists collaborated on goals, debriefed, reviewed language, and engaged in in-vivo discussions.	Parent: Separate training sess. (mediated home practice); Teacher: NR. Home-practice: Same as Parsons et al (2019a).
(Ratcliffe et al., 2014) Require purchase	EBSST (Wong et al., 2010): 16-sess. F2F school-based prog. for ASD without ID (3–8 children), delivered over 3 terms, 90 minutes/sess., with 6-mo f/up booster. Comparator: CTR. CBT & ABA: focus on emotion identification, PT, PS, ER strategies, self-monitoring, & evaluation. Tech.: social stories, VM, visual teaching tools, RP, emotion-coaching, psychoeducation, parent CBT. Behavioral strategies: deep breathing, relaxation, distraction.	School counselors; 2-day skill-based training; Online support forum; High fidelity (≥80% on self-report).	Parent: Separate training sess. (7 × 90 minutes + 1 booster at 6-mo f/up); Teachers: Separate training sess. (7 × 90 minutes + 1 booster at 6-mo f/up). HW assigned to parents and children.
(Ratcliffe et al., 2019) Require purchase	EBSST (Ratcliffe et al., 2010): 16-sess. F2F school-based prog. for ASD + MID (3–8 children), delivered over 3 terms, 60–90 minutes/sess., with a 6-mo f/up booster. Comparator: WLC. CBT & ABA: focus on ER, body signs, VPT, SPS, FCK, self-monitoring, and FP. Tech.: social stories, VM, visual teaching tools, RP, and emotion-coaching. Behavioral strategies: deep breathing, relaxation, and distraction.	School counselors (14 years avg. experience); 2-day training by authors; Online forum; High fidelity (≥80% on training assessment; 7-point Likert scale).	Parent: Separate training sess. (7 × 90 minutes + 1 booster at 6-mo f/up); Teacher: Separate training sess. (7 × 90 minutes + 1 booster at 6-mo f/up). HW & communication book.
(Salem-Guirgis et al., 2019) Contact original author	MYmind (de Bruin et al., 2015): 10-wk F2F clinic-based mindfulness-based prog. for ASD without ID (2–3 PCD); 90 minutes/sess. + 1 booster at 9-wk post-prog. No comparator; pre-post design. Mindfulness-based + CBT: focus on ER, mindfulness, and self-control for children, mindful parenting. Tech.: breathing exercises, yoga, body scanning.	CP, behavioral consultants, post-doc, clin. psych grad. students; 5-day training (MBCT/MBSR) + 3-day silent retreat; Content checklist; VR; Fidelity (80.5% fidelity, 97.3% reliability).	Parent: Separate mindfulness training sess. (concurrent sess.); Teacher: NR. Guided meditation recordings sent home for practice.
(Soorya et al., 2015) Seaver Research Center	Seaver-NETT (Soorya et al., 2015): 12-wk F2F clinic-based prog. for ASD without ID (4–6 children), 90 minutes/sess. RCT; active CTR (Facilitated play group). CBT approach: focus on nonverbal communication, emotion recognition, and ToM. Tech.: Skillstreaming, RP, visual supports.	CP, therapy assistants; High fidelity (97.4%) using treatment fidelity checklists; 3 independent raters.	Parent: Separate training sess. (concurrent parent group sess., wk. skills practice at home); Teacher: NR. Wk. skills practice, token economy system.
(Stichter et al., 2012) May need to contact the author.	SCI-E (Stichter et al., 2010): 10-wk F2F clinic-based prog. for elementary students with AS and HFASD (4–7 children); 2x/wk, 60 minutes/sess. No comparator; pre-post design. Clinic-based prog. CBT & ABA: focus on ToM, emotion recognition, and executive functioning. Tech.: RP, visual supports, structured group activities.	Master’s level educators specialized in autism; Implementers with specific training, consistent curriculum delivery, structured activities; Fidelity (92% skills, 97% activities, 89% daily notes).	Parent: Limited involvement; Teacher: In-sess. facilitation. NR.
(Tanksale et al., 2021) May need to contact the author.	Incredible Explorers (Tanksale et al., 2021): 6-wk F2F community-based prog. for self-regulation in children with ASD without ID (4–5 PCD); 60 minutes/sess. RCT; WLC. Community-based. Yoga-based: breathing exercises, mindfulness, body scanning, postures integrated with third-wave CBT elements.	CP, student volunteers; Manualized training; Supervision; Home practice logs; Fidelity (92% overall; 85%–96% across components).	Parent: In-sess. participation & separate de-briefing training; Teacher: NR. Weekly home practice, bedtime relaxation recordings.
(Thomeer et al., 2012) May need to contact the author.	Comprehensive Psychosocial Treatment (Lopata et al., 2010): 5-wk F2F clinic-based prog. for HFASD (6 children), 5 days/wk, 350 minutes/day (5x70-minute cycles). RCT; WLC. CBT & ABA: focus on social skills, ER, non-literal language, and interest expansion. Tech.: Skillstreaming, direct instruction, RP, performance feedback.	UG & grad. students; 100% fidelity on written exam; 5 days training; High fidelity (95.3% skills groups; 97.5% activities; high adherence).	Parent: Separate training sess. (90min/wk); Teacher: NR. IDN, weekly PT.
(Thomson et al., 2015) Require purchase	SAS Operation Regulation (Beaumont et al., 2015): 10-wk F2F clinic-based prog. for ER in children with ASD without ID (1 child, parent(s), therapist), 60 minutes/sess. No comparator; pre-post design. CBT approach targeting ER. Tech.: RP, multimedia activities (computer games), modeling, relaxation/mindfulness strategies.	Grad. students, post-doc fellow (supervised); High fidelity (89.6% across sess.).	Parent: In-sess. participation (provided feedback, strategies for home practice); Teacher: NR. Wk. HW & practice ER skills.

*Note*. SAS = Secret Agent Society; CSBI = Comprehensive School-Based Intervention; SCI-E = Social Competence Intervention-Elementary; Seaver-NETT = Seaver-Nonverbal Communication, Emotion Recognition, and Theory of Mind Training; EBSST = Emotion-based Social Skills Training; ABA = applied behavior analysis; CBT = cognitive-behavioral therapy; ASD = autism spectrum disorder; HFASD = high-functioning autism spectrum disorder; AS = Asperger’s Syndrome; ID = intellectual disability; MID = mild intellectual disability; OP = outpatient; SPED = special education teacher; CP = Licensed Clinical Psychologist; clin. psych = clinical psychology; OT = occupational therapist; SLP = speech-language pathologist; SWs = social workers; grad. = graduate; UG = undergraduate; CTR = control group; WLC = waitlist-control group; TAU = treatment as usual; SAU = services-as-usual; PCD = parent-child dyads; TAs = therapeutic activities; SPS = structured problem-solving; PS = problem-solving; ToM = Theory of Mind; ER = emotion regulation; PT = perspective-taking; RP = role-play; VM = video modeling; VR = video-recorded session; VPT = Visual Perspective-taking; FP = Feeling Party; FCK = Feelings Control Kit; HSD = Home-School Diary; IDN = individual daily note; HW = homework; psychoed. = psychoeducation; Tech. = technique(s); comm. = communication; comp. = comprehensive; sess. = session; mod. = module; mo = months; wk = week; f/up = follow-up; NS = not specified; TD = typically developing child.

#### Intervention Approach and Key Components

The interventions utilized a range of approaches, including Secret Agent Society (SAS; developed by Beaumont, 2009, 2010, 2015; CBT approach; *n* = 3), Emotion-based Social Skills Training (EBSST; developed by Ratcliffe et al., 2010; Wong et al., 2010; CBT + ABA approach; *n* = 2), Comprehensive School-Based Intervention-MAXout (CSBI-MAXout; developed by Lopata et al., 2010; CBT approach; *n* = 2), CSBI-SchoolMAX (developed by Lopata et al., 2010; CBT + ABA approach; *n* = 2), CSBI-Community-SummerMAX (developed by Lopata et al., 2010; CBT approach; *n* = 1), Comprehensive Psychosocial Treatment (developed by Lopata et al., 2010; CBT + ABA approach; *n* = 1), peer-to-peer play-based interventions (developed by Wilkes-Gillan et al., 2016; play-based approach; *n* = 2), MYmind (developed by de Bruin et al., 2015; mindfulness + CBT approach; *n* = 1), Seaver-Nonverbal Communication, Emotion recognition, and Theory of mind Training (Seaver-NETT; developed by Soorya et al., 2015; CBT approach; *n* = 1), Social Competence Intervention-Elementary (SCI-E; developed by Stichter et al., 2010; CBT + ABA approach; *n* = 1), and Incredible Explorers (developed by Tanksale et al., 2021; yoga + third-wave CBT approach; *n* = 1).

Some interventions, such as SAS and CSBI, have been adapted across studies to fit different contexts and populations. While SAS (Beaumont, 2009, 2010, 2013, 2015) retained its CBT framework, refinements included multimedia tools, emotion regulation techniques, and behavioral reinforcement strategies. Similarly, CSBI (Lopata et al., 2010) evolved into MAXout, SchoolMAX, and Community-SummerMAX, differing in delivery format and integration of CBT and ABA approaches.

Key components across the interventions included the use of structured activities to enhance social skills, emotion regulation, problem-solving, and adaptive behavior. Some interventions, such as the Incredible Explorers, also incorporated third-wave CBT components and yoga to target executive functioning and emotion awareness.

#### Intervention Dose, Group Size, and Delivery Mode

The duration of intervention ranged from 5 to 43 weeks, with session intensity varying between one session per week (i.e., duration and frequency; lasting 45–90 minutes) and more frequent formats, including two sessions per week (Lopata et al., 2017, 2021) or the more intensive daily sessions (Lopata et al., 2015; Thomeer et al., 2012).

Nine interventions lasted 5–10 weeks (Beaumont et al., 2015; Lopata et al., 2015; Parsons et al., 2019a, 2019b; Salem-Guirgis et al., 2019; Stichter et al., 2012; Tanksale et al., 2021; Thomeer et al., 2012; Thomson et al., 2015), one lasted 12 weeks (Soorya et al., 2015), and seven exceeded 12 weeks (Einfeld et al., 2018; Lopata et al., 2012, 2017, 2019, 2021; Ratcliffe et al., 2014, 2019).

Group sizes across the 17 studies ranged from 2 to 15 participants (Beaumont et al., 2015; Einfeld et al., 2018; Lopata et al., 2012, 2015, 2017, 2019, 2021; Parsons et al., 2019a, 2019b; Ratcliffe et al., 2014, 2019; Salem-Guirgis et al., 2019; Soorya et al., 2015; Stichter et al., 2012; Tanksale et al., 2021; Thomeer et al., 2012; Thomson et al., 2015). One study (Lopata et al., 2019) included sessions with 1 to 5 participants, classifying single-participant sessions as individual while meeting the criteria for group-based interventions. Despite variations in timing, frequency, and duration, most interventions consisted of weekly 1-hour group sessions.

The interventions were delivered face-to-face in various settings, including community centers, outpatient clinics, and educational institutions. Ten studies reported using an intervention manual for GBER delivery (Beaumont et al., 2015; Einfeld et al., 2018; Lopata et al., 2012, 2015, 2017, 2019; Parsons et al., 2019a, 2019b; Ratcliffe et al., 2014, 2019).

#### Program Facilitators, Protocol Adherence and Fidelity

Program facilitators across the interventions included clinical psychologists (*n* = 4), school staff (i.e., school counselors/guidance officers, teachers/classroom aides/teaching assistants, classroom teachers, learning support staff, special education teachers; *n* = 14), social workers (*n* = 1), speech therapists (*n* = 4), OTs (*n* = 2), and other professionals, such as graduate/post-doctoral fellows, undergraduate students, research team members, community agency staff, therapy assistants, volunteers (*n* = 10). All 17 studies reported adherence to the intervention protocol and fidelity monitoring. Various methods were employed, including the use session checklists, facilitator training, videotaped sessions and independent coding, and supervision to ensure consistent implementation.

#### Parent and/or Teacher Involvement

Parent and teacher involvement varied across studies in terms of receiving and delivering interventions. In 82% of studies, parents participated in separate training sessions to support their child’s SEC development at home (Beaumont et al., 2015; Einfeld et al., 2018; Lopata et al., 2012, 2015, 2017, 2019, 2021; Parsons et al.,2019a, 2019b; Ratcliffe et al., 2014, 2019; Salem-Guirgis et al., 2019; Soorya et al., 2015; Thomeer et al., 2012). In two studies, parents were directly involved in sessions alongside their children (Tanksale et al., 2021; Thomson et al., 2015) while one study emphasized both separate training and joint sessions for parents and children (Tanksale et al., 2021). However, one study (Stichter et al., 2012) did not specify the nature of parent involvement.

Teachers received separate training to apply intervention strategies in the classroom, in two studies (Ratcliffe et al., 2014, 2019). In five studies, teachers were actively involved during sessions, undertaking activities alongside children (Beaumont et al., 2015; Einfeld et al., 2018; Lopata et al., 2012, 2019; Stichter et al., 2012).

Out of the 17 studies, seven involved both parents and teachers, either separately or alongside children, to promote skill generalization (Beaumont et al., 2015; Einfeld et al., 2018; Lopata et al., 2012, 2019; Ratcliffe et al., 2014, 2019; Stichter et al., 2012). Most studies (88%) included homework tasks to reinforce skills outside of the sessions (Beaumont et al., 2015; Einfeld et al., 2018; Lopata et al., 2012, 2015, 2017, 2019, 2021; Parsons et al., 2019a, 2019b; Ratcliffe et al., 2014, 2019; Salem-Guirgis et al., 2019; Soorya et al., 2015; Tanksale et al., 2021; Thomson et al., 2015).

#### Accessibility

Few interventions were freely accessible or commercially available. Open access was available for only two interventions: SummerMAX and SchoolMAX accessible through their respective websites. SAS and Emotion-based Social Skill Training (EBSST, now known as the Westmead Feelings Program, WFP), were commercially available. All other interventions required direct contact with the author to obtain the manual and materials.

### Outcome Measures

A wide range of SEC and related skills were assessed, along with measures of executive functioning. See Table 4 for details. Most of the primary outcome measures for SEC were obtained through informant reports from parents, teachers, children and clinician staff ratings. None of the studies, however, assessed the degree of parent or teacher engagement during GBER interventions. No social engagement outcome measures were identified.

**Table 4:** Summary of Intervention Outcomes.

Article, intervention & critical appraisal score (JBI)	Outcome measures	Results
(Beaumont et al., 2015); SAS; 8/9	Primary: SSQ (Parent & Teacher); ERSSQ (Parent & (Teacher); Child measures of social problem-solving (James & Dylan) Secondary: SCAS-P; CAPES-DD (Parent & Teacher)	Sig. findings: Social skills, emotion regulation & problem-solving skills sig. improved in Condition 1, with gains maintained at 6-wk f/up (*p* < .001). Parent-reported anxiety levels sig. decreased (*p* < .001, *η*² = .19). Non-sig. findings: No sig. improvement in teacher-reported emotion regulation, social skills, parent-reported anxiety, or teacher-reported child behavior for Condition 2.
(Einfeld et al., 2018); SAS; 9/9	Primary: SSQ (Parent & Teacher); ERSSQ (Parent & Teacher); Child measures of social problem-solving (James & Dylan)	Sig. findings: Parent- and teacher-reported social skills, emotion regulation, and problem-solving improved, with gains sustained at 12-month f/up (*p* < .001). Non-sig. findings: No sig. changes in teacher-reported social skills/ emotion regulation post-intervention or during the waitlist period (TAU) (*p* > .05).
(Lopata et al., 2017); CSBI-MAXout; 7/9	Primary: CASL Idiomatic Language (Child); CAM-C Faces (child); ASC (Parent) & (Clinician); BASC-2 Social Skills (Parent & Clinician) & BSI (Parent & Clinician); SRS-2 (Clinician)	Sig. findings: Idiomatic language skills sig. improved (*p* < .05). Emotion recognition abilities sig. enhanced (*p* < .001). ASD-related symptoms sig. reduced in both parent and clinician ratings (*p* < .05). Clinicians reported sig (*p* < .01). Non-sig. findings: No sig. differences in social skills generalization outside clinical settings. No sig. improvement in behavioral regulation (BASC-2 BSI).
(Lopata et al., 2021); CSBI-MAXout; 9/13	Primary: SRS-2 (Parent); ASC (Parent) Secondary: CASL Idiomatic Language (Child); BASC-3 Social Skills & BSI (Parent)	Sig. findings: Social responsiveness, social skills (SRS-2, ASC), and idiomatic language (CASL) sig. improved. ASD-related behaviors and social interaction difficulties sig. reduced. Social skills and behavioral symptoms (BASC-3) also sig. improved. Non-sig. findings: No sig. improvements in other social cognition areas. Mixed results for social skills generalization. No sig. differences in BASC-3 BSI.
(Lopata et al., 2012); CSBI-SchoolMAX; 7/9	Primary: SKA (Child); CAM-C (Child); DANVA-2-Child Faces (Child) & Adult Faces (Child); ASC (Teacher & Parent); BASC-2 Social Skills (Teacher & Parent); SRS (Teacher & Parent)	Sig. findings: Children showed sig. improvements in social skills knowledge (*p* < .001, *d* = 1.31) and recognition of facial and vocal emotions (*p* < .001, *d* = 1.64). with better emotion recognition in children’s faces (*p* = .003, *d* = 1.27). Both parents and teachers reported sig. improvements in social skills, with stronger effects noted by parents (ASC: *p* < .001, *d* = 1.22; BASC-2: *p* = .007, d = 0.75) compared to teachers (ASC: *p* = .014, *d* = 0.59; BASC-2: *p* = .037, *d* = 0.57). Non-sig. findings: No sig. improvements were found for recognizing emotions in adult faces (*p* = .150, *d* = 0.45) or teacher-rated reductions in ASD-related symptoms (SRS: *p* = .190, *d* = 0.19). However, parents reported sig. reductions in ASD symptoms (SRS: *p* = .003, *d* = 0.92).
(Lopata et al., 2019); CSBI-SchoolMAX; 8/13	Primary: CAM-C (Child); SRS-2 (Parent-Teacher Rating) Secondary: ASC (Parent); SIOS: Behavioral Observation; WJ-III Ach: Academic Skills	Sig findings: Emotion recognition skills improved sig. in the CSBI group compared to the SAU control group (CAM-C: *p* < .001, *d* = 1.41). ASD symptoms sig. reduced as rated by parents and teachers in the CSBI group (SRS-2: *p* < .001, *d* = −1.15), alongside improvement in social & communication skills in the CSBI group (ASC: *p* = .001, *d* = 1.29). Non-sig. findings: No sig. differences in positive social interactions during recess (SIOS: *p* = .692, *d* = .08), nor in academic skills across all WJ-III Ach subtests.
(Lopata et al., 2015); CSBI-Community-SummerMAX; 8/9	Primary: CASL Idiomatic Language (Child); DANVA-2 Child Faces (Child); ASC (Parent & Staff); BASC-2 Social Skills (Parent & Staff) & Withdrawal (Parent & Staff); SRS (Parent & Staff)	Sig. findings: Idiomatic Language skills sig. increased (CASL: *p* < .001, *d* = 0.52). ASC ratings and social skills sig. improved for parents (*p* < .001, *d* = 0.63) and staff (*p* < .001, *d* = 1.40). Social skills improved sig. for both parents (*p* = .009, *d* = 0.27) and staff (*p* < .001, *d* = 0.80), with reductions in SRS symptoms for parents (*p* < .001, *d* = 0.46) and staff (*p* < .001, *d* = 0.74). Non-sig. findings: No sig. improvement in emotion recognition (DANVA-2 Child Faces: *p* = .072, *d* = 0.18) or reduction in BASC-2 Withdrawal for parents (*p* = .125, *d* = 0.23).
(Parsons et al., 2019a); Peer-to-peer play-based intervention; 7/9	Primary: SEE (Clinician); POM (Clinician)	Sig. findings: TD-playmates sig. improved in pragmatic language (POM: *p* = .011) and social-emotional skills (SEE: *p* = .035) with gains maintained at 3-month f/up. Non-sig. findings: No sig. differences between groups or across environments.
(Parsons et al., 2019b); Peer-to-peer play-based intervention; 12/13	Primary: SEE (Clinician); POM (Clinician)	Sig. findings: Social communication and pragmatic language skills improved in autistic children (*p* < .001), with gains maintained at 3-month f/up. Non-sig. findings: No sig. differences between groups for baseline variables or changes in SEE scores between post-intervention and 3-month follow-up for TD-playmates.
(Ratcliffe et al., 2014); EBSST; 8/9	Primary: EDQ (Teacher & Parent); SSIS (Teacher & Parent) Secondary: DBC (Teacher & Parent); SDQ (Teacher & Parent)	Sig. findings: Emotional competence sig. improved in the EBSST group compared to control group (EDQ-T/P: *p* < .0001, *η²* = 0.18), with gains maintained at 6-month f/up. Non-sig. findings: No sig. improvements in social skills (SSIS-T/P) or mental health (DBC or SDQ: SDQ-T *p* = .995; SDQ-P *p* = .214). Clinically significant teacher-reported SDQ improvements (“high” to “borderline” difficulties) were not maintained at 6-month f/up.
(Ratcliffe et al., 2019); EBSST; 8/9	Primary: EDQ (Teacher & Parent); SSIS (Teacher & Parent) Secondary: DBC (Teacher & Parent)	Sig. findings: Emotional competence sig. improved in the EBSST group (EDQ-T/P), but gains were not sustained at a 6-month f/up. Adjustments with Bonferroni adjustment (*p* > .05). Non-sig. findings: No sig. improvements in social skills (SSIS-T/P) or mental health (DBC).
(Salem-Guirgis et al., 2019); MYmind; 8/9	Primary: BASC-2 SRP (Youth) & PRS (Parent); ERC (Youth); ERQ-CA (Youth); SRS-2 (Parent); RRS (Parent) Secondary: CAMM; DASS-21 (Parent); FFMQ-SF (Parent); IEM-P (Parent)	Sig. findings: Youth adaptive skills (BASC-2), reflection (RRS), autism symptoms (SRS-2), and restrictive/repetitive behaviors (SRS-2 RRBs) sig. improved (*p* < .05), with parent mindfulness (FFMQ-SF) also improving post-program and at f/up. Non-sig. findings: No sig. changes in youth-reported internalizing problems (BASC-2 SRP), mindfulness (CAMM), expressive suppression (ERQ-CA), or parent mental health (DASS-21).
(Soorya et al., 2015); Seaver-NETT; 11/13	Primary: SRS (Parent); Griffith Empathy Measure (Parent); CCC-2 (Parent); DANVA-2 (Clinician); RMET (Clinician); Strange Stories Task (Clinician)	Sig. findings: Social behavior (e.g., nonverbal communication & empathy) sig. improved in the NETT group compared to control (SRS: *p* = .04, *d* = 0.88). Post hoc analysis showed improvements in identifying low-intensity emotions on (DANVA2: *p* < .01, *d* = 0.56). Non-sig. findings: No sig. improvements in global social cognition composite or differences between groups at 3-month f/up.
(Stichter et al., 2012); SCI-E; 7/9	Primary: DANVA-2 Child Facial Expressions; Reading in mind’s eye, Faux Pas stories; ToM: Sally Anne & Smarties false belief task; BRIEF (Parent); TOPS-3 (Clinician); SRS (Parent & Teacher)	Sig. findings: Social cognition (*p* < .001, *d* = 0.61), social communication (*p* < .001, *d* = 0.74), and overall social abilities (*p* < .001, *d* = 0.75) improved sig. Recognition of social faux pas improved (*p* < .01, d = 0.40), global executive functioning (*p* < .01, d = 0.35), & metacognition (*p* < .01, *d* = 0.42) improved. Non-sig. findings: No sig. improvement in emotional/mental states identification. Mixed ToM results with some regression. Only TOPS-3 sequencing scale showed sig. improvement.
(Tanksale et al., 2021); Incredible Explorers; 8/13	Primary: Executive functions (BRIEF-2): BRI, ERI, CRI (Parent); EAQ Secondary: CHSQ (Parent); ASC-ASD (Children); GAS	Sig. findings: Executive functions sig. improved post-intervention (BRIEF-2: *p* = .047, *d* = −0.39) and at follow-up (*p* = .017, *d* = −0.59). Sig. improvements were also found in sleep (*p* = .024, *d* = −0.48) and emotional awareness (*p* = .005, *d* = 0.59), with performance anxiety sig. reduced at follow-up (*p* = .028, *d* = −0.43). Non-sig. findings: No sig. changes were reported in other anxiety subscales or emotion regulation measures.
(Thomeer et al., 2012); CSBI-SummerMAX; 9/13	Primary: ASC (Parent); SRS (Parent); BASC-2 PRS; SKA (Child); DANVA-2 Child Faces (Child); CASL Idiomatic Language (Child)	Sig. findings: Social skills sig. improved (ASC: *p* < .001, *d* = 1.57; BASC-2 Social Skills: *p* = .003, *d* = .84), along with reductions in ASD-related symptoms (SRS: *p* = .001, *d* = .86). Emotion knowledge also improved (SKA: *p* < .001, *d* = 1.15). Gains were maintained at follow-up for ASC (*p* = .006, *d* = .47) and BASC-2 Social Skills (*p* = .004, *d* = .68). Non-sig. findings: No sig. improvement in facial emotion recognition (DANVA-2: *p* = .078) or BASC-2 Withdrawal (*p* = .087).
(Thomson et al., 2015); SAS; 7/9	Primary: (ERC Lability/Negativity, Emotion Regulation (Parent); ADIS-P-IV; BASC-2 Externalizing Behaviors, Internalizing Behaviors, BSI, Adaptive Behavior (Parent); CEMS-Inhibition, Dysregulation, Coping (Child); CGI Scale Secondary: Scenarios James and the Math Test, Dylan is Being Teased (Clinician)	Sig. findings: Parent-reported improvements in emotional lability, internalizing symptoms, dysregulation, and adaptive behavior (*p* < .05). Clinician-rated severity and diagnoses (ADIS-P-IV) improved, and children reported better coping strategies post-intervention. Non-sig. findings: No sig. change in the CGI-S scores for illness severity.

*Note.* SAS = Secret Agent Society; CSBI = Comprehensive School-Based Intervention; EBSST = Emotion-based Social Skills Training; Seaver-NETT = Seaver-Nonverbal Communication, Emotion Recognition, and Theory of Mind Training; SCI-E = Social Competence Intervention-Elementary; SSQ = Social Skills Questionnaire; SSQ-P = Social Skills Questionnaire—Parent; SSQ-T = Social Skills Questionnaire—Teacher; ERSSQ = Emotion Regulation Skills Scale Questionnaire; SCAS-P = The Spence Children’s Anxiety Scale—Parent Version; CAPES-DD-P = Child Adjustment and Parent Efficacy Scale-Developmental Disability—Parent; CAPES-DD-T = Child Adjustment and Parent Efficacy Scale-Developmental Disability—Teacher; CASL = Comprehensive Assessment of Spoken Language; CAM-C = Cambridge Mindreading Face-Voice Battery for Children; ASC = Adapted Skillstreaming Checklist; BASC-2 = Behavior Assessment System for Children, Second Edition; BASC-2 BSI = Behavioral Symptoms Index for BASC-2; BASC-2 SRP = Self-Report of Personality for BASC-2; BASC-2 PRS = Parent Rating Scales for BASC-2; BASC-3 = Behavior Assessment System for Children, Third Edition; SRS = Social Responsiveness Scale; SRS-2 = Social Responsiveness Scale, Second Edition; SKA = Skillstreaming Knowledge Assessment; DANVA-2 = Diagnostic Analysis of Nonverbal Accuracy, Second Edition; SIOS = Social Interaction Observation Scale; WJ-III Ach = Woodcock-Johnson III Tests of Achievement; SEE = Social Emotional Evaluation; POM = Pragmatics Observation Measure; POM-2 = Pragmatics Observation Measure, Second Edition; EDQ-P = Emotion Development Questionnaire—Parent; EDQ-T = Emotion Development Questionnaire—Teacher; SSIS-P = Social Skills Improvement System—Parent; SSIS-T = Social Skills Improvement System—Teacher; DBC-P = Developmental Behavior Checklist—Parent; DBC-T = Developmental Behavior Checklist—Teacher; SDQ = Strengths and Difficulties Questionnaire; ERQ-CA = Emotion Regulation Questionnaire for Children and Adolescents; RRS = Ruminative Response Scales; CAMM = Child and Adolescent Mindfulness Measure; DASS-21 = Depression, Anxiety, and Stress Scale; FFMQ-SF = Five Facets of Mindfulness Questionnaire-Short Form; IEM-P = Interpersonal Mindfulness in Parenting Scale; CCC-2 = Children’s Communication Checklist, Version 2; RMET = Reading the Mind in the Eyes Test; ToM = Theory of Mind; BRIEF = Behavior Rating Inventory of Executive Function; BRIEF-2 = Behavior Rating Inventory of Executive Function, Second Edition; BRI = Behavioral Regulation Index for BRIEF-2; ERI = Emotional Regulation Index for BRIEF-2; CRI = Cognitive Regulation Index for BRIEF-2; TOPS-3 = Test of Problem Solving, Third Edition; EAQ = Emotion Awareness Questionnaire; CHSQ = Children’s Sleep Habits Questionnaire; ASC-ASD = Anxiety Scale for Children–Autism Spectrum Disorder; GAS = Goal Attainment Scale; ERC = Emotion Regulation Checklist; ADIS-P-IV = Anxiety Disorder Interview Schedule: Parent Interview- 4th Edition; CEMS = Children’s Emotion Management Scale; CGI = Clinical Global Impressions Scale; Sig. = significant; Non-sig. = non-significant; wk. = week; f/up = follow-up; TAU = Treatment-as-usual; ASD = Autism Spectrum Disorder; TD = typically developing.

#### Socio-Emotional Competence

All studies included measured SEC as a primary outcome. The most frequently used tools were the Behavior Assessment System for Children-2 (BASC-2; Reynolds & Kamphaus, 2004; *n* = 5), Diagnostic Analysis of Nonverbal Accuracy-2 (DANVA-2; Nowicki, 1997; *n* = 5), Social Responsiveness Scale (SRS; Constantino & Gruber, 2005; *n* = 5), Social Responsiveness Scale-2 (SRS-2; Constantino, 2012; *n* = 4), and Comprehensive Assessment of Spoken Language (CASL; Carrow-Woolfolk, 1999; *n* = 4).

Other SEC measures included the Emotion Development Questionnaire (EDQ; Wong et al., 2009; *n* = 2), Social Skills Improvement System (SSIS; Gresham & Elliott, 2008; *n* = 2), Skillstreaming Knowledge Assessment (SKA; Goldstein et al., 1997; *n* = 2), Emotion Regulation and Social Skills Questionnaire (ERSSQ; Beaumont & Sofronoff, 2008; *n* = 2), Emotion Regulation Checklist (ERC; Shields & Cicchetti, 1997; *n* = 2), James and the Maths Test/Dylan Is Being Teased (Attwood, 2004a, 2004b; *n* = 3), Social Skills Questionnaire (SSQ; Spence, 1995; *n* = 2), Griffith Empathy Measure (Dadds et al., 2008; *n* = 1), Children’s Communication Checklist-2, Version 2 (CCC-2; Bishop, 2003; *n* = 1), Reading the Mind in the Eyes Test (RMET; Baron-Cohen et al., 2001; *n* = 1), Strange Stories Task (Brent et al., 2004; *n* = 1), Theory of Mind tasks: Sally Anne and Smarties false belief (Baron-Cohen et al., 1985; Perner et al., 1989; *n* = 1), Children’s Emotion Management Scale (CEMS; Zeman et al., 2001, 2010; *n* = 1), and Test of Problem Solving-3 (TOPS-3; Bowers et al., 2005; *n* = 1).

#### Executive Functioning

Executive functions were measured alongside SEC, using tools such as the Behavior Rating Inventory of Executive Function (BRIEF; Gioia et al., 2000; *n* = 1) and Behavior Rating Inventory of Executive Function-2 (BRIEF-2; Gioia et al., 2015; *n* = 1).

#### Other Functional Outcome Measures

Several studies included secondary outcome measures, assessing areas such as anxiety reduction, overall psychological well-being, academic skills, mindfulness and goal-setting. The tools used to measure these outcomes included Children’s Sleep Habits Questionnaire (CHSQ; Owens et al., 2000; *n* = 1), Anxiety Scale for Children—for Children Autism Spectrum Disorder—self-report (ASC-ASD; Rodgers et al., 2016; *n* = 1), Anxiety Disorders Interview Schedule: Parent Interview—4th Edition (ADIS-P-IV; Silverman & Albano, 1996; *n* = 1), Developmental Behavior Checklist (DBC; Einfeld & Tonge, 1992; *n* = 1), Strengths and Difficulties Questionnaire (SDQ; Goodman, 1997; *n* = 1), Child and Adolescent Mindfulness Measure (CAMM; Greco et al., 2011; *n* = 1), Woodcock-Johnson III Tests of Achievement (WJ-III Ach; Woodcock et al., 2001; *n* = 1), and Goal Attainment Scale (GAS; Kiresuk & Sherman, 1968; *n* = 1).

### Risk of Bias Assessment Scores

The methodological quality of the included studies varied. For quasi-experimental studies, scores ranged from 7/9 to 9/9, with a median score of 7 points using the JBI Critical Appraisal Checklist for Quasi-Experimental Studies (Tufanaru et al., 2020). Studies scoring 8 or 9 were classified as low risk of bias. For RCTs, scores ranged from 8/13 to 12/13, with a median score of 9 points using the JBI Critical Appraisal Checklist for Randomized Controlled Trials (Tufanaru et al., 2020). Studies scoring 10 or higher were considered low risk of bias. Transparent reporting of risk of bias scores is critical for ensuring reproducibility and clarity in systematic reviews (Viswanathan et al., 2017). See Supplemental Tables 2 and 3 for specific scores assigned to all included studies. Three of the eight studies rated in the low-risk-of-bias range—SAS (Beaumont et al., 2015), Community-SummerMAX (Lopata et al., 2015), and MYmind (Salem-Guirgis et al., 2019)—did not include a comparator group.

### Effectiveness of GBER Intervention(s)

Due to heterogeneity of the included studies, a meta-analysis was not feasible (see Table 4). However, all studies reported positive trends for primary outcome measures, although the specific focus and significance varied. Among the eight low-risk-of-bias studies, two were RCTs (Parsons et al., 2019b; Soorya et al., 2015), and six were quasi-experimental (Beaumont et al., 2015; Einfeld et al., 2018; Lopata et al., 2015; Ratcliffe et al., 2014, 2019; Salem-Guirgis et al., 2019). These studies evaluated six interventions: SAS, Community-SummerMAX, peer-to-peer play-based intervention, EBSST, MYmind, and Seaver-NETT.

Significant improvements in SEC were observed across studies, particularly in social skills, emotional competence, and empathy. Large effect sizes were reported for social skills in SAS and Community-SummerMAX, while EBSST demonstrated moderate improvements in emotional competence. Notable gains in nonverbal communication and pragmatic language were also recorded in peer-to-peer play-based interventions. Improvements in parent mindfulness were documented in MYmind, though no teacher-related outcomes were explicitly reported. For detailed statistics, including effect sizes, refer to Table 4.

## Discussion

This systematic review examined the effectiveness and characteristics of GBER interventions on SEC in autistic children with mild or no ID, aged 7 to 18 years. A total of 17 studies (*n* = 1,218 children) were included, with six interventions identified across eight studies categorized as low risk of bias. These interventions are discussed in detail below.

### Effectiveness of GBERs

All six GBER interventions were effective in improving SEC, although their specific areas of focus varied across social, emotional, and other skills. The greatest improvement in social skills was observed in the SAS and Community-SummerMAX interventions. Moderate gains in emotional competence were observed for EBSST, while empathy and pragmatic language improved through Seaver-NETT and peer-to-peer play-based interventions. Further, parent mindfulness also improved in the MYmind. These findings support the broader application of group-based approaches to enhance access for the autistic population. However, several studies lacked comparator groups, making it difficult to attribute SEC improvements solely to interventions, as other factors may have influenced the outcomes.

### Characteristics GBERs

The six most effective interventions shared key characteristics that contributed to their success. All interventions were based on cognitive-behavioral therapy (CBT), focusing on emotion regulation and social skills. Specific features of each intervention included play-based models, such as peer-to-peer interventions, to improve pragmatic language. Psychoeducation for parents and teachers, central to SAS, Community-SummerMAX, EBSST, and MYmind, enables them to reinforce skills at home and school environments. Mindfulness training was emphasized in MYmind, while structured homework tasks reinforced skills across interventions.

#### Key Components

CBT components such as psychoeducation, self-awareness, behavioral strategies, and cognitive restructuring were present in all interventions. Programs such as SAS and EBSST provided structured parental emotion-coaching, focusing on emotion regulation and social skills.

MYmind included mindfulness activities, like breathing exercises and yoga, while other interventions incorporated interactive tools (e.g., worksheets, computer games, visual supports). Despite their success, however, both goal-setting and behavioral activation received less emphasis, although they have the potential to enhance parent engagement with children and sustain implementation. Addressing these gaps may increase the overall effectiveness of interventions by reinforcing parent involvement at home.

#### Parent and/or Teacher Involvement

Active parent and teacher involvement was prioritized in five interventions (SAS, Community-SummerMAX, EBSST, MYmind, and peer-to-peer play-based intervention), but not in Seaver-NETT. Although the importance of such involvement was well emphasized by Ratcliffe et al. (2019), none of the interventions formally measured the degree of parent/teacher engagement during sessions. Future research could explore this engagement using tools such as the Pediatric Rehabilitation Intervention Measure of Engagement—Parent version (PRIME-P; King et al., 2022) or the Observation version (PRIME-O). These tools may assist in assessing active participation and optimistic outcomes.

#### Structured Homework

Structured homework tasks were implemented in all six interventions. Homework implementation was reported in eight studies, although its direct correlation with outcomes was not evaluated. Studies like SAS and MYmind for instance, used diaries and guided meditation to reinforce learning. Future studies should measure homework adherence to assess its role in improving intervention success.

#### Dose

Interventions doses varied, typically between 60 and 90 minutes per session over 9–12 weeks. Parental and/or teacher training sessions in SAS, EBSST, and Community-Summer MAX, ranged from 90 minutes to 2 hours. Three interventions (MYmind, EBSST, and SAS) offered booster sessions at follow-ups of 9 weeks, 6 months, or 12 months. Higher-dose interventions, such as SAS, Community-SummerMAX, and EBSST, generally yielded greater effects than lower-dose ones, such as peer-to-peer play-based intervention. Although no consistent dose-effect relationship was identified, combining higher doses with intensive parent and/or teacher involvement and non-clinic-based settings (e.g., community- or school-based programs) tended to yield significant outcomes (Beaumont et al., 2015; Einfeld et al., 2018; Lopata et al., 2015; Ratcliffe et al., 2014, 2019). Future research should explore optimal doses for SEC improvement.

#### Delivered by

All interventions were designed to be delivered by a range of trained facilitators including school staff, clinical psychologists, OTs, and speech therapists. The Community-SummerMAX program required a multidisciplinary team, including psychologists and speech-language pathologists, highlighting the need for specialized expertise for some interventions. However, the flexibility of delivery across professions demonstrates the broad applicability of GBERs.

#### Facilitator’s Training & Fidelity

All six interventions were manualized, with structured facilitator training ensuring fidelity. Training was delivered through various approaches including in-person and online formats, ranging from one-time workshops to episodic five-day sessions. This emphasis on facilitator fidelity supports the consistent delivery of GBERs across diverse professionals, maintaining both quality and consistency in interventions while benefiting from the unique expertise of different disciplines. For example, OTs have a strong tradition of client-centered care, where the perspectives, values and priorities of the client are central to all therapeutic processes (Townsend & Polatajko, 2013). In the context of GBERs for families with autistic children, OTs are attuned to the responses of children and parents during learning activities, actively eliciting and integrating their experiences into interventions.

In addition, OTs’ orientation on enabling occupations in life roles and routines emphasizes occupational outcomes, such as personally valued goals expressed through the “doing” of child, parent and family occupations (Mandich & Rodger, 2006; Rodger & Ziviani, 2006). OTs’ expertise in occupational analysis (Chapparo, 2010; Mills & Chapparo, 2017) informs tailored responses to parents’ challenges, such as implementing homework or resolving challenges in managing daily routines with children. Similarly, other professionals will bring their unique perspectives to the delivery of GBERs, while maintaining fidelity to the core intervention components.

#### Outcomes

Individualized goal-setting, as an outcome measure, could provide valuable insights into perceived impact of GBERs on the lived experience of families. However, none of the eight studies classified as low risk of bias evaluated the impact of these interventions on individualized goals for autistic children, their parents, or teachers. One study with a higher risk of bias (Incredible Explorers) included individualized goals as an outcome measure. Measuring individualized, personally meaningful goals is central to both occupational therapy and family-centered practice (Chien et al., 2020; Graham et al., 2020) making it relevant to interventions designed to support children and families. Goal-setting is a key component of CBT—the foundation of most/all of the six interventions classified as low risk of bias identified in this review. Research has demonstrated that collaboratively developed goals with parents positively impact client engagement and behavior activation (i.e., implementation of strategies with children) (Brewer et al., 2014). Several tools—such as Canadian Occupational Performance Measure (COPM; M. Law et al., 1991), Goal-Based Outcomes (GBO; D. Law, 2011), and Goal Attainment Scaling (GAS; Kiresuk & Sherman, 1968)—could identify and measure individualized goals in GBER interventions research. However, it is unclear which of these tools would fit best with the group-based delivery format of GBERs, particularly given their focus on emotional competence skills rather than goals related to life situations or specific occupations.

#### Mode of Delivery

All 17 included studies were delivered face-to-face. Only one study (Ratcliffe et al., 2019) suggested telehealth delivery (e.g., app/iPad) as a potential avenue for future research, although this format was not evaluated in any of the interventions reviewed. Evidence from telehealth-delivered CBT indicates its effectiveness in addressing conditions such as childhood posttraumatic stress, insomnia symptoms, and psychiatric disorders (e.g., depressive symptoms, chronic pain, generalized anxiety disorder) (Kautzmann et al., 2023; Matsumoto et al., 2021; Stewart et al., 2020). These telehealth CBT approaches commonly involve structured session plans, guided by standardized intervention protocols, and include key components such as psychoeducation and therapist fidelity checks. Given the similarities between components and those in SEC-focused interventions, telehealth delivery could potentially benefit children’s SEC as well. Feasibility studies are necessary to confirm whether SEC improvement, particularly with parent and teacher involvement, could be effectively replicated through telehealth-delivered GBER interventions.

#### Cross Cultural Considerations

Implementing GBER interventions in diverse cultural contexts may require thoughtful adaptation to meet the unique needs of specific populations. While many recent studies reviewed have predominantly focused on White/Caucasian populations, it is important to recognize that most interventions were developed and tested in Western settings with limited representation from other racial and ethnic groups (Lopata et al., 2015; Salem-Guirgis et al., 2019; Soorya et al., 2015; Thomeer et al., 2012). Expanding these interventions to African, Latin American, Asian, and Indigenous populations, would require culturally responsive adaptations that address varying socio-cultural norms and linguistic nuances (Hernandez et al., 2020; Ivanich et al., 2018; Ward et al., 2022). Balancing GBER interventions’ fidelity with flexibility for cultural adaptation is crucial to ensure that interventions remain effective, acceptable, and relevant in diverse cultural settings (Lee et al., 2023a, 2023b). Future research should clarify what tailoring is needed for GBER interventions in culturally diverse populations while maintaining their efficacy.

### Implications for Clinical Practice and Future Research

The review highlights several key implications. First, GBER interventions offer an effective alternative to individualized therapy, enabling scalability and broader access to families and groups. Second, future research could explore the feasibility and effectiveness of extending the reach of GBER interventions through telehealth delivery and cross-cultural tailoring. Third, OTs may be well-positioned to deliver existing GBER interventions, supported with facilitation training, manuals, and delivery resources. Finally, applying an occupational therapy lens to existing GBER interventions may enhance their delivery through inclusion of individualized and occupation-based goals in GBER interventions research.

### Limitations

The potential limitation of this systematic review is the inability to locate all available evidence, as gray literature (e.g., online documents) was not included. Some studies lacked comparison groups, making it difficult to conclude that GBERs led to the observed changes in children’s SEC. Due to the heterogeneity of the included studies, a meta-analysis was not feasible, and findings are presented as a narrative synthesis. The search was limited to specific databases and time frames, and terms such as “telehealth” and “COVID-19” were not included in the initial search strategy. However, such studies would have been identified if they met other inclusion criteria. Future reviews could examine all telehealth-delivered interventions addressing SEC, which may have implications for group-based delivery.

## Conclusions

This systematic review identified six GBER interventions categorized as low risk of bias—Community-SummeMAX, EBSST, MYmind, peer-to-peer play-based intervention, SAS, and Seaver-NETT—that improve SEC in autistic children, particularly emotion regulation, social communication, empathy, and pragmatic language. CBT-based approaches were the most prevalent, together with play-based and mindfulness elements incorporated in GBER interventions. However, the lack of comparator groups in several studies limits the conclusiveness of these findings. Despite this, GBER interventions have potential to improve the reach of interventions aimed at supporting both autistic children’s SEC and parental goals and well-being.

OTs may be well-positioned to deliver several manualized GBER interventions, further enhancing intervention outcomes through their expertise in client-centered and occupation-focused goal-setting. Future research should aim to clarify appropriate individualized outcome measures and explore the adaptation of GBER interventions to telehealth delivery formats and in more diverse cultural contexts.

## Supplemental Material

sj-docx-1-otj-10.1177_15394492251330507 – Supplemental material for Systematic Review of Group-Based Emotion Regulation Interventions for Autistic Children’s Socio-Emotional Competence Supplemental material, sj-docx-1-otj-10.1177_15394492251330507 for Systematic Review of Group-Based Emotion Regulation Interventions for Autistic Children’s Socio-Emotional Competence by Sitii Hazwaanii Jasni, Fiona Graham, Elliot Bell and Valerie T.Y. Tan in OTJR: Occupational Therapy Journal of Research

sj-docx-2-otj-10.1177_15394492251330507 – Supplemental material for Systematic Review of Group-Based Emotion Regulation Interventions for Autistic Children’s Socio-Emotional Competence Supplemental material, sj-docx-2-otj-10.1177_15394492251330507 for Systematic Review of Group-Based Emotion Regulation Interventions for Autistic Children’s Socio-Emotional Competence by Sitii Hazwaanii Jasni, Fiona Graham, Elliot Bell and Valerie T.Y. Tan in OTJR: Occupational Therapy Journal of Research

sj-docx-3-otj-10.1177_15394492251330507 – Supplemental material for Systematic Review of Group-Based Emotion Regulation Interventions for Autistic Children’s Socio-Emotional Competence Supplemental material, sj-docx-3-otj-10.1177_15394492251330507 for Systematic Review of Group-Based Emotion Regulation Interventions for Autistic Children’s Socio-Emotional Competence by Sitii Hazwaanii Jasni, Fiona Graham, Elliot Bell and Valerie T.Y. Tan in OTJR: Occupational Therapy Journal of Research

sj-docx-4-otj-10.1177_15394492251330507 – Supplemental material for Systematic Review of Group-Based Emotion Regulation Interventions for Autistic Children’s Socio-Emotional Competence Supplemental material, sj-docx-4-otj-10.1177_15394492251330507 for Systematic Review of Group-Based Emotion Regulation Interventions for Autistic Children’s Socio-Emotional Competence by Sitii Hazwaanii Jasni, Fiona Graham, Elliot Bell and Valerie T.Y. Tan in OTJR: Occupational Therapy Journal of Research
