# Supplementary material for: Systematic Review of Group-Based Emotion Regulation Interventions for Autistic Children’s Socio-Emotional Competence
Source: OTJR (Thorofare N J). 2025 Apr 15;46(3):402–21. doi: 10.1177/15394492251330507 (PMC13219792; doi:10.1177/15394492251330507)
Supplement: sj-docx-1-otj-10.1177_15394492251330507 – Supplemental material for Systematic Review of Group-Based Emotion Regulation Interventions for Autistic Children’s Socio-Emotional Competence [file sj-docx-1-otj-10.1177_15394492251330507.docx]

**Supplemental Table 1**

*Search Strategy and Results*

| **No** | **Database** | **Keywords/Search strategy** | **Result** |
| --- | --- | --- | --- |
| 1 | Scopus | ( ABS ( autis* OR asperger* OR "pervasive developmental disorder" OR "Autism Spectrum Disorder" ) AND ABS ( child* OR pediatric* OR paediatric* OR teen* OR adoles* ) AND ABS ( "emotion* focused" OR "emotion* control" OR "emotion* regulation" OR "emotion* dysregulation" OR "mood regulation" OR "affect* regulation" OR "emotion* intelligence" OR "emotion* competence" OR "emotion* based" OR "emotion* control" OR mindful* OR "social-emotional" OR "emotion-based social skills" OR feeling* OR "emotion* development" OR "emotion* recognition" OR "emotion* awareness" ) AND ABS ( intervention* OR treatment* OR module* OR program* OR training* OR learning* ) AND ABS ( parent* OR mother* OR father* OR caregiver* OR famil* OR home* ) AND ABS ( teach* OR educat* OR "schoolteacher*" OR tutor* OR coach* OR train* OR instruct* OR mentor* OR "school counsellor*" OR school* OR "teach* aide*" ) ) AND PUBYEAR > 2011 AND PUBYEAR < 2023 | 241 |
| 2 | Web of Science | autis* OR asperger* OR "pervasive developmental disorder" OR "Autism Spectrum Disorder'" (Abstract) AND child* OR pediatric* OR paediatric* OR teen* OR adoles* (Abstract) AND "emotion* focused" OR "emotion* control" OR "emotion* regulation" OR "emotion* dysregulation" OR "mood regulation" OR "affect* regulation" OR "emotion* intelligence" OR "emotion* competence" OR "emotion* based" OR "emotion* control" OR mindful* OR "social-emotional" OR "emotion-based social skills" OR feeling* OR "emotion* development" OR "emotion* recognition" OR "emotion* awareness" (Abstract) AND intervention* OR treatment* OR module* OR program* OR training* OR learning* (Abstract) AND parent* OR mother* OR father* OR caregiver* OR famil* OR home* (Abstract) AND teach* OR educat* OR "schoolteacher*" OR tutor* OR coach* OR train* OR instruct* OR mentor* OR "school counsellor*" OR school* OR "teach* aide*" (Abstract) | 144 |
| 3 | MEDLINE (Ovid) | ((autis* or asperger* or "pervasive developmental disorder" or "Autism Spectrum Disorder") and (child* or pediatric* or paediatric* or teen* or adoles*) and ("emotion* focused" or "emotion* control" or "emotion* regulation" or "emotion* dysregulation" or "mood regulation" or "affect* regulation" or "emotion* intelligence" or "emotion* competence" or "emotion* based" or "emotion* control" or mindful* or "social-emotional" or "emotion-based social skills" or feeling* or "emotion* development" or "emotion* recognition" or "emotion* awareness") and (intervention* or treatment* or module* or program* or training* or learning*) and (parent* or mother* or father* or caregiver* or famil* or home*) and (teach* or educat* or "schoolteacher*" or tutor* or coach* or train* or instruct* or mentor* or "school counsellor*" or school* or "teach* aide*")).ab.  limit 1 to yr="2012 -Current"5. | 128 |
| 4 | PsycInfo (Ovid) | ((autis* or asperger* or "pervasive developmental disorder" or "Autism Spectrum Disorder") and (child* or pediatric* or paediatric* or teen* or adoles*) and ("emotion* focused" or "emotion* control" or "emotion* regulation" or "emotion* dysregulation" or "mood regulation" or "affect* regulation" or "emotion* intelligence" or "emotion* competence" or "emotion* based" or "emotion* control" or mindful* or "social-emotional" or "emotion-based social skills" or feeling* or "emotion* development" or "emotion* recognition" or "emotion* awareness") and (intervention* or treatment* or module* or program* or training* or learning*) and (parent* or mother* or father* or caregiver* or famil* or home*) and (teach* or educat* or "schoolteacher*" or tutor* or coach* or train* or instruct* or mentor* or "school counsellor*" or school* or "teach* aide*")).ab.  limit 1 to yr="2012 – 2023 | 48 |
| 5 | PubMed | (((((autis*[Title/Abstract] OR asperger*[Title/Abstract] OR "pervasive developmental disorder"[Title/Abstract] OR "Autism Spectrum Disorder"[Title/Abstract]) AND (child*[Title/Abstract] OR pediatric*[Title/Abstract] OR paediatric*[Title/Abstract] OR teen*[Title/Abstract] OR adoles*[Title/Abstract])) AND ("emotion* focused"[Title/Abstract] OR "emotion* control"[Title/Abstract] OR "emotion* regulation"[Title/Abstract] OR "emotion* dysregulation"[Title/Abstract] OR "mood regulation"[Title/Abstract] OR "affect* regulation"[Title/Abstract] OR "emotion* intelligence"[Title/Abstract] OR "emotion* competence"[Title/Abstract] OR "emotion* based"[Title/Abstract] OR mindful*[Title/Abstract] OR "social-emotional"[Title/Abstract] OR "emotion-based social skills"[Title/Abstract] OR feeling*[Title/Abstract] OR "emotion* development"[Title/Abstract] OR "emotion* recognition"[Title/Abstract] OR "emotion* awareness"[Title/Abstract] OR)) AND (intervention*[Title/Abstract] OR treatment*[Title/Abstract] OR module*[Title/Abstract] OR program*[Title/Abstract] OR training*[Title/Abstract] OR learning*[Title/Abstract])) AND (parent*[Title/Abstract] OR mother*[Title/Abstract] OR father*[Title/Abstract] OR caregiver*[Title/Abstract] OR famil*[Title/Abstract] OR home*[Title/Abstract])) AND (teach*[Title/Abstract] OR educat*[Title/Abstract] OR "schoolteacher*"[Title/Abstract] OR tutor*[Title/Abstract] OR coach*[Title/Abstract] OR train*[Title/Abstract] OR instruct*[Title/Abstract] OR mentor*[Title/Abstract] OR "school counsellor*"[Title/Abstract] OR school*[Title/Abstract] OR "teach* aide*"[Title/Abstract]) | 127 |
| 6 | CINAHL | "AB ( autis* OR asperger* OR "pervasive developmental disorder" OR "Autism Spectrum Disorder" ) AND AB ( child* OR pediatric* OR paediatric* OR teen* OR adoles* ) AND AB ( "emotion* focused" OR "emotion* control" OR "emotion* regulation" OR "emotion* dysregulation" OR "mood regulation" OR "affect* regulation" OR "emotion* intelligence" OR "emotion* competence" OR "emotion* based" OR "emotion* control" OR mindful* OR "social-emotional" OR "emotion-based social skills" OR feeling* OR "emotion* development" OR "emotion* recognition" OR "emotion* awareness" ) AND AB ( intervention* OR treatment* OR module* OR program* OR training* OR learning* ) AND AB ( parent* OR mother* OR father* OR caregiver* OR famil* OR home* ) AND AB ( teach* OR educat* OR "schoolteacher*" OR tutor* OR coach* OR train* OR instruct* OR mentor* OR "school counsellor*" OR school* OR "teach* aide*" ) | 70 |
| 7 | Complete Psychology & Behavioural Sciences Collection (Ebsco) | "AB ( autis* OR asperger* OR "pervasive developmental disorder" OR "Autism Spectrum Disorder" ) AND AB ( child* OR pediatric* OR paediatric* OR teen* OR adoles* ) AND AB ( "emotion* focused" OR "emotion* control" OR "emotion* regulation" OR "emotion* dysregulation" OR "mood regulation" OR "affect* regulation" OR "emotion* intelligence" OR "emotion* competence" OR "emotion* based" OR "emotion* control" OR mindful* OR "social-emotional" OR "emotion-based social skills" OR feeling* OR "emotion* development" OR "emotion* recognition" OR "emotion* awareness" ) AND AB ( intervention* OR treatment* OR module* OR program* OR training* OR learning* ) AND AB ( parent* OR mother* OR father* OR caregiver* OR famil* OR home* ) AND AB ( teach* OR educat* OR "schoolteacher*" OR tutor* OR coach* OR train* OR instruct* OR mentor* OR "school counsellor*" OR school* OR "teach* aide*" ) Published Date: 20120101-20221231 | 172 |
| **Total** | | | **930** |
