# Supplementary material for: Systematic Review of Group-Based Emotion Regulation Interventions for Autistic Children’s Socio-Emotional Competence
Source: OTJR (Thorofare N J). 2025 Apr 15;46(3):402–21. doi: 10.1177/15394492251330507 (PMC13219792; doi:10.1177/15394492251330507)
Supplement: sj-docx-2-otj-10.1177_15394492251330507 – Supplemental material for Systematic Review of Group-Based Emotion Regulation Interventions for Autistic Children’s Socio-Emotional Competence [file sj-docx-2-otj-10.1177_15394492251330507.docx]

**Supplemental Table 2**

*Risk of Bias Assessment Scores for Randomized Controlled Trials (RCTs)*

| **Randomized Controlled Trials (RCTs)*** | **1.** | **2.** | **3.** | **4.** | **5.** | **6.** | **7.** | **8.** | **9.** | **10.** | **11.** | **12.** | **13.** | **JBI Score** |
| --- | --- | --- | --- | --- | --- | --- | --- | --- | --- | --- | --- | --- | --- | --- |
| Lopata et al. (2021)  CSBI-MAXout | + | ? | + | - | - | - | + | + | + | + | + | + | + | 9 |
| Lopata et al. (2019)  CSBI-SchoolMAX | + | ? | + | ? | - | - | ? | + | + | + | + | + | + | 8 |
| Parsons et al. (2019b) Peer-to-peer play-based intervention | + | + | + | + | + | + | + | + | ? | + | + | + | + | 12 |
| Soorya et al. (2015) Seaver-NETT | + | + | + | - | - | + | + | + | + | + | + | + | + | 11 |
| Tanksale et al. (2021) Incredible Explorers | + | - | + | - | - | - | - | + | + | + | + | + | + | 8 |
| Thomeer et al. (2012) SummerMAX | + | ? | + | - | - | - | + | + | + | + | + | + | + | 9 |
| **Questions:**  1. Was true randomization used for assignment of participants to treatment groups?  2. Was allocation to treatment groups concealed?  3. Were treatment groups similar at the baseline?  4. Were participants blind to treatment assignment?  5. Were those delivering treatment blind to treatment assignment?  6. Were outcomes assessors blind to treatment assignment?  7. Were treatment groups treated identically other than the intervention of interest?  8. Was follow up complete and if not, were differences between groups in terms of their follow up adequately described and analyzed?  9. Were participants analyzed in the groups to which they were randomized?  10. Were outcomes measured in the same way for treatment groups?  11. Were outcomes measured in a reliable way?  12. Was appropriate statistical analysis used?  13. Was the trial design appropriate, and any deviations from the standard RCT design (individual randomization, parallel groups) accounted for in the conduct and analysis of the trial   \| **Indicator:**  + low risk  - high risk  ? unclear risk \| \| --- \| | | | | | | | | | | | | | | |

*Note.* CSBI = Comprehensive School-Based Intervention; Seaver-NETT = Seaver-Nonverbal Communication, Emotion recognition, and Theory of mind Training. *Based on JBI Critical Appraisal Checklist for Randomised Controlled Trials (Tufanaru et al., 2020).

**Supplemental Table 3**

*Risk of Bias Assessment Scores for* *Quasi-Experimental Studies*

| **Quasi-Experimental Studies*** | **1.** | **2.** | **3.** | **4.** | **5.** | **6.** | **7.** | **8.** | **9.** | **JBI Score** |
| --- | --- | --- | --- | --- | --- | --- | --- | --- | --- | --- |
| Beumont et al. (2015)  SAS | + | + | + | - | + | + | + | + | + | 8 |
| Einfeld et al. (2018) SAS | + | + | + | + | + | + | + | + | + | 9 |
| Lopata et al. (2017) CSBI-MAXout | + | + | + | - | + | - | + | + | + | 7 |
| Lopata et al. (2015) CSBI-Community-SummerMAX | + | + | + | - | + | + | + | + | + | 8 |
| Lopata et al. (2012) CSBI-SchoolMAX | + | + | - | - | + | + | + | + | + | 7 |
| Parsons et al. (2019a) Peer-to-peer play-based intervention | + | + | - | - | + | + | + | + | + | 7 |
| Ratcliffe et al. (2019) EBSST | + | + | - | + | + | + | + | + | + | 8 |
| Ratcliffe et al. (2014) EBSST | + | + | - | + | + | + | + | + | + | 8 |
| Salem-Guirgis et al. (2019) MYmind | + | + | + | - | + | + | + | + | + | 8 |
| Stichter et al. (2012) SCI-E | + | + | ? | - | + | + | + | + | + | 7 |
| Thomson et al. (2015) SAS | + | + | + | - | + | - | + | + | + | 7 |
| **Questions**   1. Is it clear in the study what is the ‘cause’ and what is the ‘effect’? 2. Were the participants included in any comparisons similar? 3. Were the participants included in any comparisons receiving similar treatment/care, other than the exposure or intervention of interest? 4. Was there a control group? 5. Were there multiple measurements of the outcome both pre and post the intervention/exposure? 6. Was follow up complete and if not, were differences between groups in terms of their follow up adequately described and analyzed? 7. Were the outcomes of participants included in any comparisons measured in the same way? 8. Were outcomes measured in a reliable way? 9. Was appropriate statistical analysis used?  \| **Indicator:**  + low risk  - high risk  ? unclear risk \| \| --- \| | | | | | | | | | | |

*Note.* SAS = Secret Agent Society (SAS); EBSST = Emotion-based Social Skills Training; SCI-E = Social Competence Intervention-Elementary. *Based on JBI Critical Appraisal Checklist for Quasi-Experimental Studies (Tufanaru et al., 2020).
